# Origami-based self-folding of co-cultured NIH/3T3 and HepG2 cells into 3D microstructures

**DOI:** 10.1038/s41598-018-22598-x

**Published:** 2018-03-14

**Authors:** Qian He, Takaharu Okajima, Hiroaki Onoe, Agus Subagyo, Kazuhisa Sueoka, Kaori Kuribayashi-Shigetomi

**Affiliations:** 1 Institute for the Advancement of Higher Education, Hokkaido University, Sapporo, Japan; 2 Graduate School of Information Science and Technology, Hokkaido University, Sapporo, Japan; 3 Department of Mechanical Engineering, Faculty of Science and Technology, Keio University, Tokyo, Japan; 4 Creative Research Institution Sousei, Hokkaido University, Sapporo, Japan

## Abstract

This paper describes an origami-inspired self-folding method to form three-dimensional (3D) microstructures of co-cultured cells. After a confluent monolayer of fibroblasts (NIH/3T3 cells) with loaded hepatocytes (HepG2 cells) was cultured onto two-dimensional (2D) microplates, degradation of the alginate sacrificial layer in the system by addition of alginate lyase triggered NIH/3T3 cells to self-fold the microplates around HepG2 cells, and then 3D cell co-culture microstructures were spontaneously formed. Using this method, we can create a large number of 3D cell co-culture microstructures swiftly with ease in the same time. We find that HepG2 cells confined in the 3D cell co-culture microstructures have an ability to enhance the secreted albumin compared to 2D system in a long culture period. The result indicates that the origami-based cell self-folding technique presented here is useful in regenerative medicine and the preclinical stage of drug development.

## Introduction

A challenge for regenerative medicine and drug development is to fabricate *in vitro* 3D structures that mimic tissues *in vivo*. Several strategies have been developed to fabricate *in vitro* 3D cell-laden structures using a bottom-up technique^1–6^, which involves micro-sized 3D cell-laden microstructures such as blocks^2^, fibers^4–6^ and spheroids^3,7^. This approach allows one to control the size and shape of these microstructures, so that they can be easily handled and assembled to mimic *in vivo* tissue.

**Figure 1:**
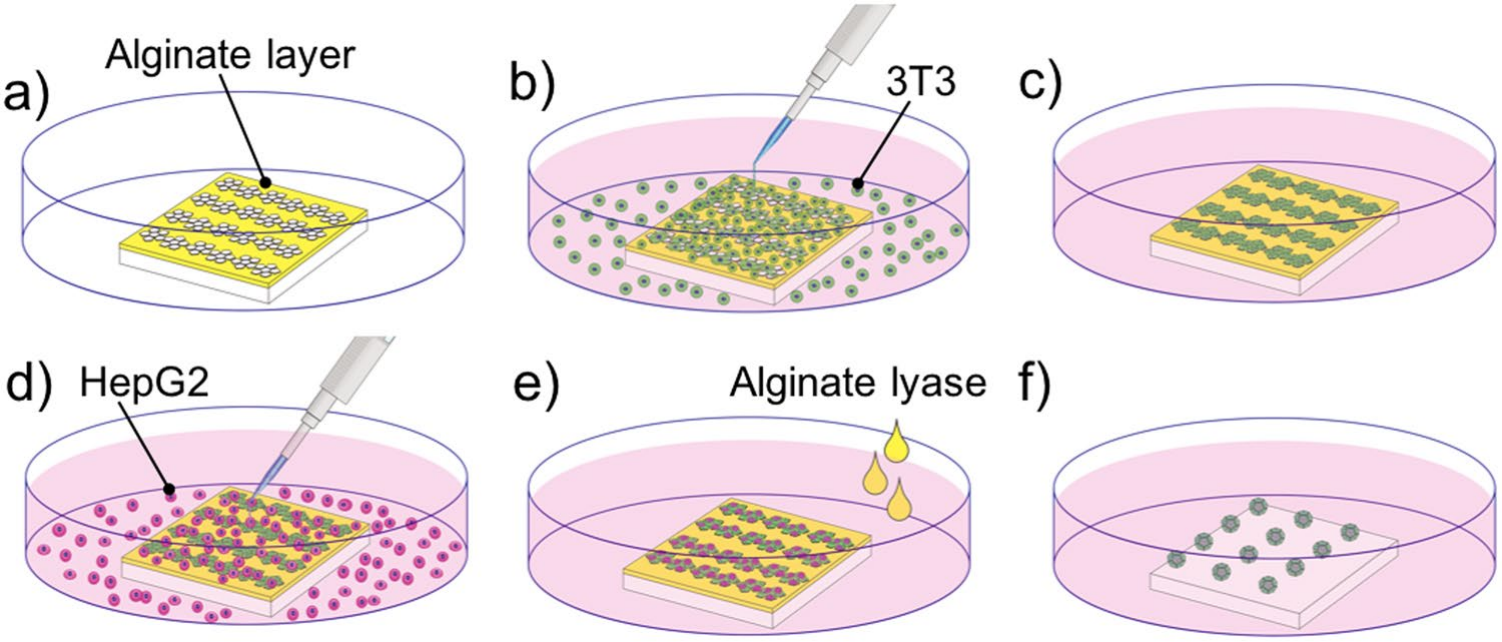
Processes of seeding and culturing cells on the microplates. (**a**) The glass substrate with microplates was placed in a petri dish. (**b**) NIH/3T3 cells were seeded on the microplates, and non-adherent cells were washed away. (**c**) Adherent NIH/3T3 cells were cultured for 24 h. (**d**) HepG2 cells were then seeded onto plates and non-adherent cells were washed away. (**e**) The attached HepG2 cells were cultured 4 h on the NIH/3T3 cells which loaded on the microplates. (**e**,**f**) After adding alginate lyase, the microplates were folded, and a number of 3D cell co-culture microstructures were formed.

3D microstructures with different types of cells have been intensively investigated to mimic *in vivo* tissues with a heterogeneous structure^3,8–11^. In this research, we applied an origami based-technique called cell origami^12^ to produce many 3D cell co-culture microstructures swiftly with ease at the same time. The process of producing 3D cell co-culture microstructures using the cell origami is as simple as that for conventional cell culture in 2D dishes (Fig. 1). The cells are grown on engineered microplates fixed to a flat surface. The microplates are then detached from the surface by degrading an alginate sacrificial layer under the plates using alginate lyase. This allows the cells to pull the plates using their traction force and self-fold around other types of cells and create a 3D culture condition. Unlike other techniques such as microfluidic devices, any extra equipment including tubes and micro pumps, is not necessary in the cell origami technique.

Other advantages of using the cell origami technique for forming 3D cell co-culture microstructures are that it can provide both flat and 3D culture conditions depending on the cell types and increase the area of interaction between co-culture cells. No other technique with these advantages has been previously developed. It is important to consider different culture conditions to retain the functions of different cell types during co-culture^13,14^. Previous researches showed that fibroblasts and endothelial cells can proliferate and retain their function on a flat substrate. Conversely, hepatocytes and pancreatic cells prefer 3D culture conditions such as in spheroids. It has also shown that interactions between different types of cells facilitates an increase in their functions^4,15–18^. A successful co-culture technique, therefore, requires the ability to i) culture one type of cell on a flat substrate, ii) culture another type of cell in 3D conditions, and iii) provide sufficient interactions between these two types of cells. These can be achieved using the cell origami technique.

Here, we produced the 3D cell co-culture microstructures with fibroblasts (NIH/3T3) and hepatoma cells (HepG2) simply and rapidly using the cell origami technique. This 3D cell co-culture microstructure provides both flat and 3D culture conditions for NIH/3T3 and HepG2 cells, respectively. We then performed a viability assay and examined the hepatic function of the co-culture cells in the 3D microstructures by analysis of secreted albumin.

## Results and Discussion

### Determination of initial NIH/3T3 cell concentration

**Figure 2:**
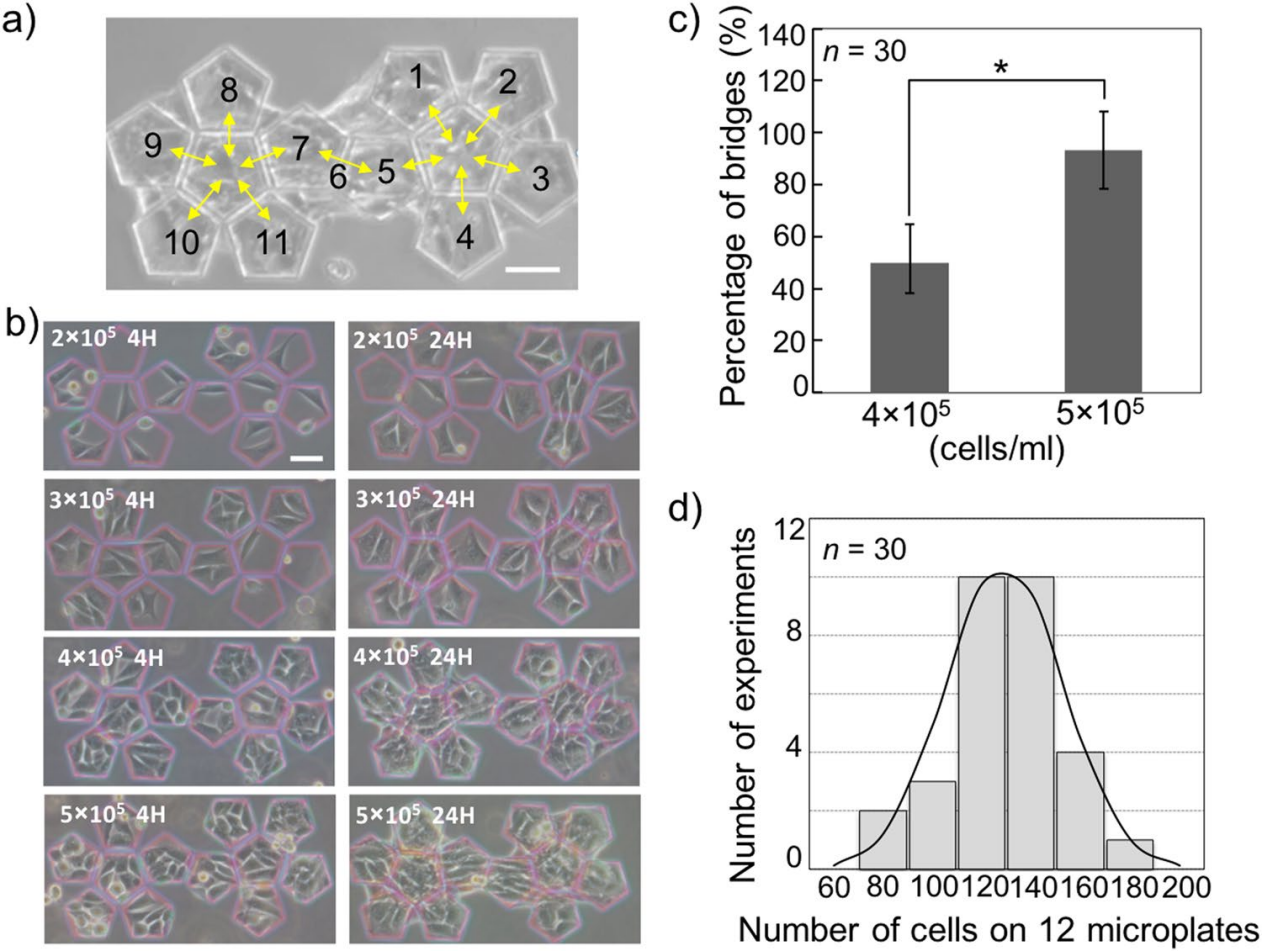
Determination of *C*_N_. (**a**) In this research, one unit included 12 pieces of microplates to form a 3D dodecahedron microstructure. The total area of each unit is 0.0516 mm^2^. The bridges of cells between the neighbouring microplates are shown with the direction of traction force by the arrows. (**b**) Examination of the occupied condition of the unit after seeding various *C*_N_ at 4 h and 24 h cultivation. (**c**) Quantification of the number of cell bridges after 24 h with *C*_N_ of 4 × 10^5^ and 5 × 10^5^ cells/ml. *p < 0.005. (**d**) After seeding 5 × 10^5^ cells/ml, the numbers of NIH/3T3 cells were counted on each unit after 24 h, scale bar = 50 µm.

To wrap HepG2 cells completely, two conditions are required for NIH/3T3 cells. First, the NIH/3T3 cells have to bridge the neighbouring microplates (depicted by the arrows in Fig. 2a) in order to behave as hinges and fold the microplates by their traction force^12^. Second, NIH/3T3 cells have to be cultured in a confluent monolayer. Thus, we first determined the initial NIH/3T3 cell concentration, *C*_N_, for satisfying these conditions.

We observed that the *C*_N_ values of 2 × 10^5^ cells/ml and 3 × 10^5^ cells/ml were insufficient for the cells to bridge the neighbouring microplates after 24 h (Fig. 2b). However, with *C*_N_ of 4 × 10^5^ cells/ml, half of the bridge regions were occupied by the cells, and with *C*_N_ of 5 × 10^5^ cells/ml, almost all of the bridge regions were occupied by the cells (Fig. 2b,c). When *C*_N_ was 5 × 10^5^ cells/ml, we found that the cells occupied all microplates as a confluent monolayer within 24 h (Fig. 2b) and that the average number of cells was approximately 130 after 24 h (Fig. 2d). Therefore, we used *C*_N_ of 5 × 10^5^ cells/ml as an optimized condition to ensure a quick formation of bridges and confluent monolayers on the microplates. Using this method, the cell numbers on the microplates could be easily controlled.

### Formation of 3D cell co-culture microstructures

**Figure 3:**
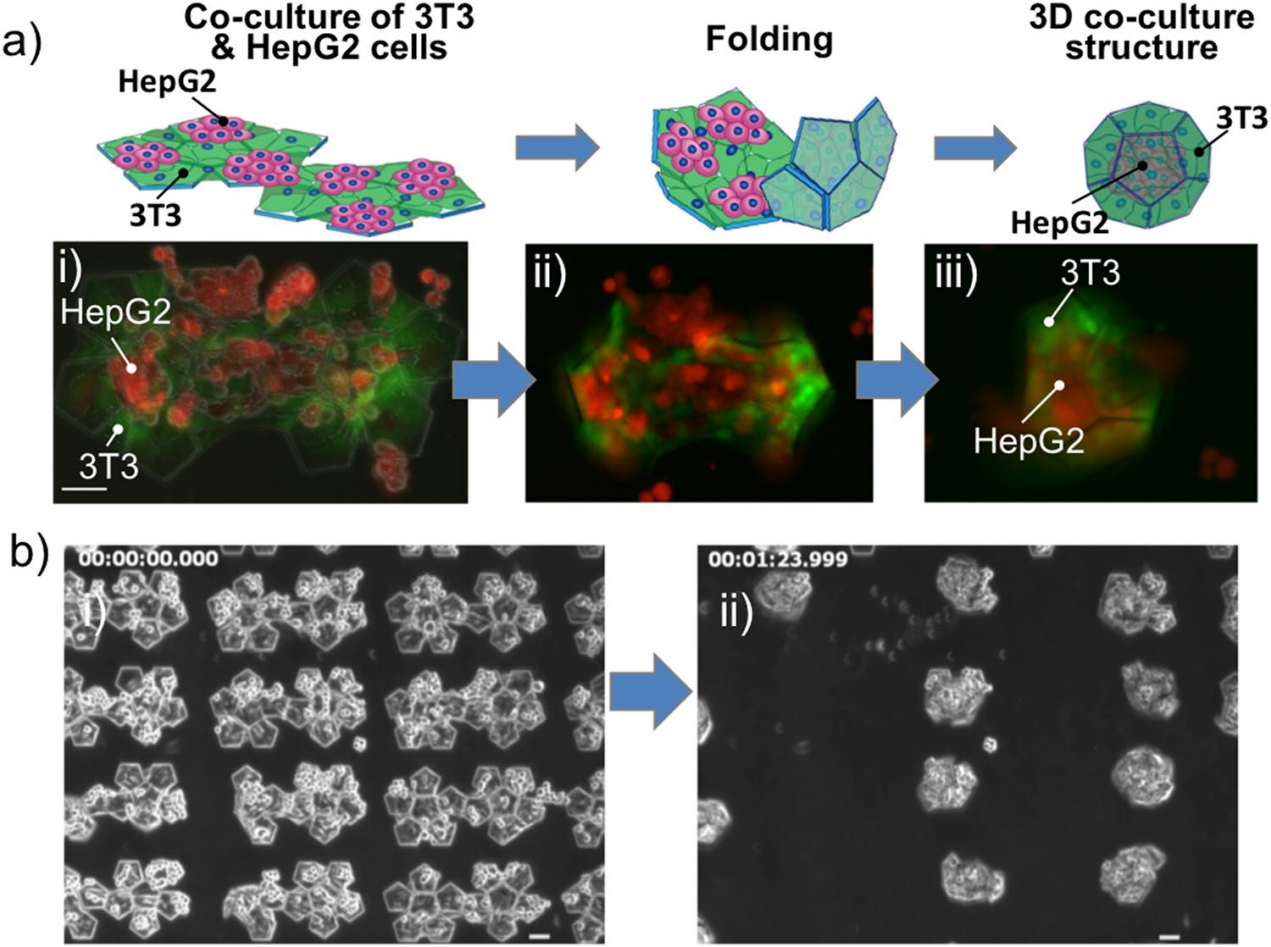
Formation of 3D cell co-culture microstructures. (**a**) Top, schematic of folding process. Bottom, NIH/3T3 and HepG2 cells dyed by CellTracker™ Green CMFDA Dye and CellTracker™ Red CMTPX Dye, respectively, were seeded on and folded the plates. (**a**-i) NIH/3T3 and HepG2 cells cultured on microplates. (**a**-ii) After addition of alginate lyase, the microplates began to fold by cell traction force. (**a**-iii) The 3D microstructure is eventually formed with the HepG2 cells covered by NIH/3T3 cells inside. (**b**) The formation of many 3D microstructures within 2 min are detected by microscopy after the addition of alginate lyase, scale bar = 50 µm.

Figure 3a–i shows an image of the HepG2 cells (red) and the NIH/3T3 cells (green) on the microplates after 4 h and 28 h culturing, respectively. NIH/3T3 cells adhered and proliferated on all areas of the microplates, and HepG2 cells were on the top of NIH/3T3 cells. As the alginate layer was degreased by the addition of alginate lyase, the microplates started to self-fold by the traction force of NIH/3T3 cells (Fig. 3a–ii, a–iii and b–i). Many dodecahedrons in which NIH/3T3 cells covered HepG2 cells were formed simultaneously (Fig. 3b–ii, Supplementary Movie 1). The self-folding process was completed within 2 min, which is much faster than 3 days in the previous study using gelatine as a sacrificial layer^12^. Our present results demonstrate that the use of the alginate layer with the cell origami folding technique can simply and rapidly create a large number of 3D cell co-culture microstructures.

**Figure 4:**
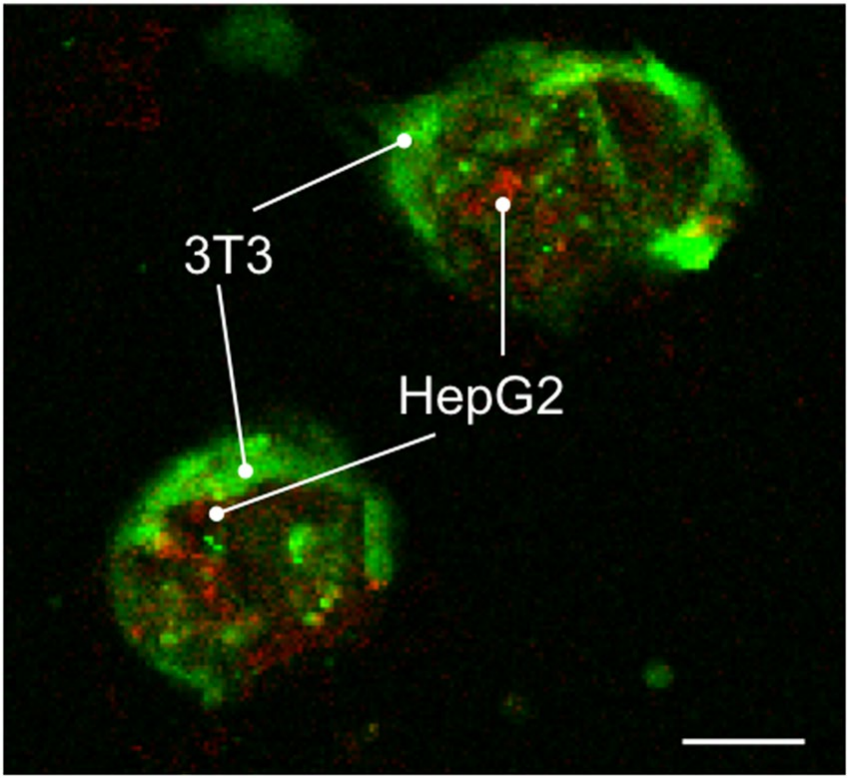
Co-cultured cells inside 3D microstructures after 3 days. HepG2 cells covered by NIH/3T3 cells were detected inside of dodecahedrons at 3 days after initial generation of microstructures, scale bar = 50 µm.

Confocal microscopic images show that HepG2 cells (red) were covered by NIH/3T3 cells (green) inside the dodecahedron after 3 days incubation in the folded state (Fig. 4, Supplementary Movie 2). This result suggested that the co-culture technique using the cell origami method is suitable for culturing one type of cell (NIH/3T3) on a flat substrate, culturing another type of cell (HepG2) in the 3D conditions, and providing sufficient area of interactions between these two types of cells.

### Viability of the co-culture cells inside the 3D microstructures

**Figure 5:**
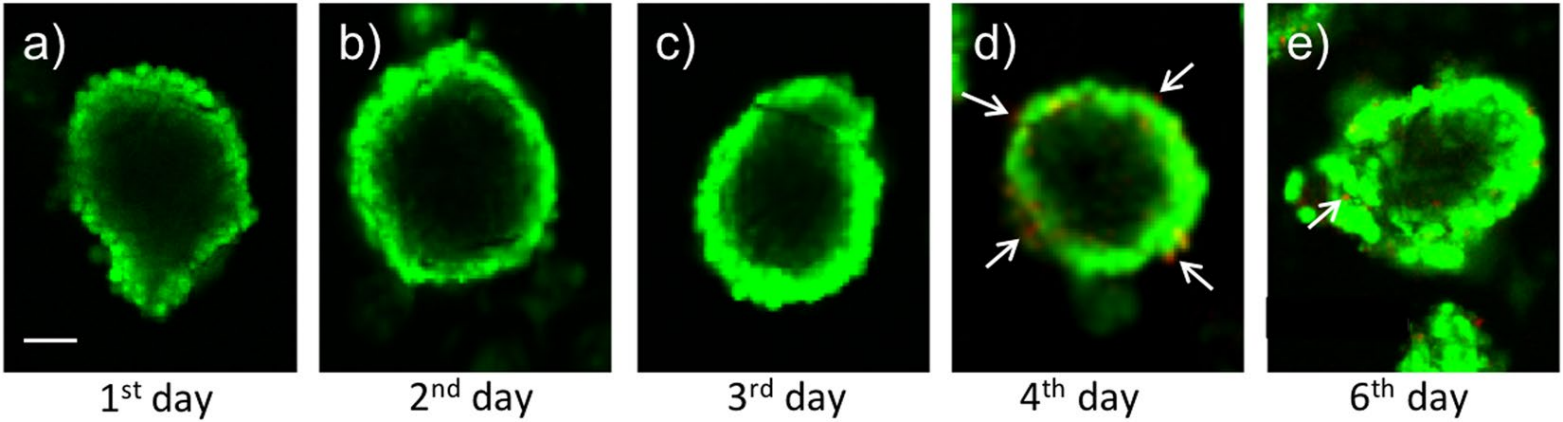
Viability of cells inside of 3D microstructures. Cells were stained by the LIVE/DEAD Viability/Cytotoxicity kit, and living and dead cells were distinguished by green and red colours, respectively. (**a**–**c**) The co-cultured cells inside 3D microstructures were alive during the first 3 days. (**d**) On the 4^th^ day, several dead cells were detected (arrows). (**e**) At the 6^th^ day, most of cells were still alive, with a few dead cells detectable (arrow), scale bar = 50 µm.

We investigated the viability of co-culture cells embedded in the microstructures. Figure 5 shows the representative images of co-culture cells inside the dodecahedrons from the 1^st^ day to the 6^th^ day. During the first three days (Fig. 5a–c), all the cells inside survived (green), indicating that NIH/3T3 and HepG2 cells were not damaged during the folding process. From the 4^th^ day to the 6^th^ day, most of the co-culture cells remained alive inside the dodecahedrons (Fig. 5d,e), even though a few dead cells (red) were observed (Fig. 5d,e, arrows). This result suggests that cells inside the 3D microstructures can survive at least 6 days. This may be because the gaps of 5 μm between the microplates allowed the medium and necessary nutrition to pass into the structures and reach the cells inside, supporting cell survival.

### Albumin secretion of co-culture cells inside the 3D microstructures

**Figure 6:**
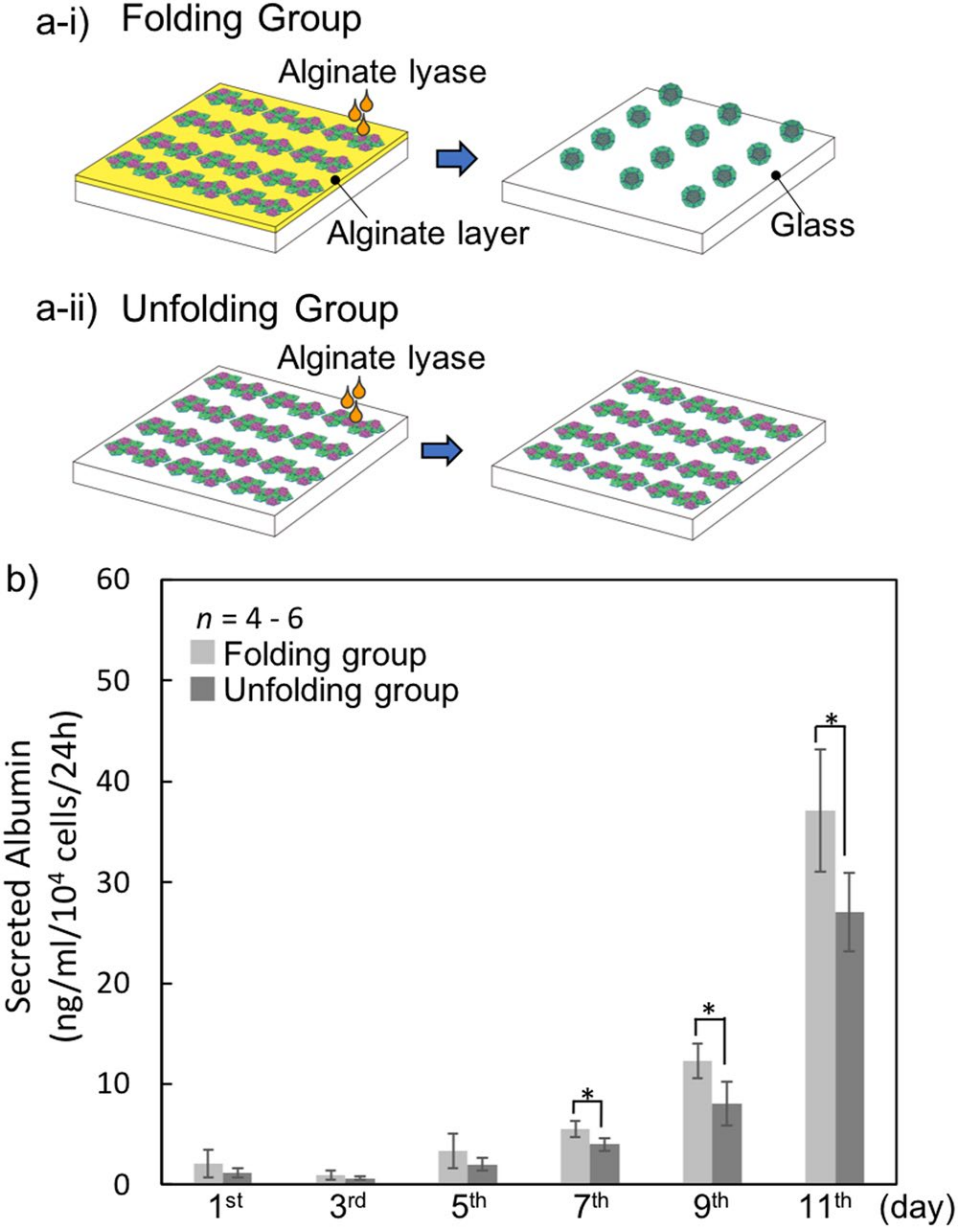
Secretion of albumin in folding and unfolding groups. (**a**-i) Folding group: microplates with alginate sacrificial layer. After adding alginate lyase, the microplates were detached from the glass substrate and folded to form a number of dodecahedrons with co-cultured cells. (**a**-ii) Unfolding group: microplates without the alginate sacrificial layer. After adding alginate lyase, the microplates could not undergo folding. (**b**) Secreted albumin levels were measured in the folding and unfolding groups from the 1^st^ day to the 11^th^ day after co-culture. *C*_N_ and *C*_H_ were 5 × 10^5^ cells/ml. Levels in each group were measured as described in Methods. *p < 0.05.

To assess the function of HepG2 cells under these culture conditions, we measured the amounts of secreted albumin from the co-culture cells in two groups, named the folding group (Fig. 6a–i) and the unfolding group (Fig. 6a–ii). The folding group is that in which the microplates with alginate layer were detached from the glass substrate and folded to form a number of dodecahedrons with co-cultured cells. The unfolding group is that in which the microplates without the alginate layer could not fold.

We next investigated how the initial HepG2 cell concentration, *C*_H_, affected the secreted albumin in the co-culture system. We observed that the secreted albumin in both folding and unfolding groups at *C*_H_ = 5 × 10^5^ cells/ml was much higher than that at *C*_H_ = 1 × 10^5^ cells/ml on the 5^th^ and 7^th^ days (Supplementary Figure 3). For *C*_H_ = 5 × 10^5^ cells/ml, the secreted albumin in both groups increased with increasing culture days (Fig. 6b). Thus, we adopted *C*_H_ = 5 × 10^5^ cells/ml for the co-culture with NIH/3T3 cells using the cell origami technique. Using this *C*_H_ condition, we found that the folding caused significantly higher albumin secretion compared with the unfolding from the 7^th^ day to the 11^th^ day (*p* < 0.05) (Fig. 6b). In the folding group, HepG2 cell clusters were surrounded by NIH/3T3 cells inside the dodecahedrons, and the cells proliferated and were tightly packed in the confined conditions. This indicates that the 3D microstructure may provide a large contact area between these two kinds of cells. In contrast, in the unfolding group, HepG2 cells formed a spheroidal structure on the NIH/3T3 cell monolayer, which reduced the contact area between HepG2 and NIH/3T3 cells and thereby restricted the cell-cell interaction between these cells. Previous studies have established that increasing cell-cell interactions between hepatocytes and feeder cells such as NIH/3T3 cells leads to increased function of hepatocytes^18,19^. Therefore, the large contact areas in the folding group may be one of the important factors that increase the function of HepG2 cells.

## Conclusion

Our findings demonstrate that many 3D cell co-culture microstructures with NIH/3T3 cells covering HepG2 cells were rapidly with ease produced by the cell origami technique using an alginate sacrificial layer. This 3D cell co-culture microstructure provides both flat and 3D culture conditions for NIH/3T3 and HepG2 cells, respectively, with sufficient interactions between these two types of cells. We confirmed that the cells survived inside of the 3D microstructures and showed that the albumin secretion function of HepG2 cells co-cultured with NIH/3T3 cells inside of the 3D microstructures was increased in comparison with cells co-cultured on the microplates. Our findings suggest that these 3D cell co-culture microstructures that have maintained the cellular functions can be applied not only in regenerative medicine and cell therapy but also in the preclinical stage of drug development.

The cell origami technique can also be applied to the production of 3D microstructures with various shapes and cell types. The shapes of the microplates can easily be varied by changing the glass masks with different designs, allowing for easy changes to the shapes of the microstructures. Human tissues that comprise different types of cells show various 3D shapes *in vivo*, and thus the cell origami technique may be useful for producing artificial human tissues that are more similar to human tissue structures *in vivo*.

## Materials and Methods

### Fabrication of cell origami substrate

Two types of microplates were prepared: microplates with and without a sacrificial layer. We used alginate as a sacrificial layer to accelerate the cell self-folding speed. Alginate degrades immediately upon addition of the alginate lyase with no damage to cells; this strategy has been used extensively for many cell-based applications^20,21^.

To form the alginate layer, 10–15 mg/ml sodium alginate (Wako, Japan) was spin-coated at 3000 rpm on the hydrophilic surface of glass substrates (20 × 20 mm, MATSUNAMI GLASS IND., LTD., Japan). The substrates were then dipped into 22 mg/ml calcium chloride (CaCl_2,_ Sigma-Aldrich, USA) solution to create the alginate layer on the glass substrates by the reaction between sodium alginate and Ca^2+^.

Parylene C (Speedline Technologies, USA) was used to produce the microplates because of its biocompatibility and ease of microfabrication^22^. The transparency property of the parylene is also useful for allowing observation of the co-cultured cells inside the 3D microstructures. Parylene microplates were made according to the previously described method^12^. Although microelectromechanical systems (MEMS) techniques are needed to fabricate the microplates for the cell origami technique, only the standard MEMS techniques are required. Briefly, the parylene layer (3 μm thickness) was deposited on the glass substrates with or without the alginate layer by chemical vapour deposition (LABCOTERPDS2010, Specialty Coating Systems, USA). Aluminium (Al) was evaporated (EBX-8C, ULVAC, Japan) on the top of the parylene (Supplementary Figure 1a). Al was patterned to define the region of the microplates using a photolithographic technique (Supplementary Figure 1b,c). The parts of exposed parylene were then removed by O_2_ plasma (10 ml/min, 25 W, RIE-10NR, SAMCO, Japan) to make the microplates (Supplementary Figure 1d).

The 2-methacryloyloxyethyl phosphorylcholine (MPC) polymer, which exhibits inhibitory properties against protein and cell adsorption, was applied to the glass substrate to pattern cells only on microplates. MPC polymer was spin-coated on the glass substrate with microplates at 2000 rpm and dried in an ethanol atmosphere chamber for 20 min to uniformly form the polymer layer. The substrate was baked at 70 °C for 4 h to form a covalent linkage between the MPC polymer and the glass surface by a dehydration reaction (Supplementary Figure 1e). Before culturing cells, the MPC polymer on the microplates was lifted-off by removing the patterned Al using an alkaline solution of NMD-3 developer (Tokyo Ohka, Japan), revealing the final parylene microplates (Supplementary Figure 1f).

Our previous study showed that we can easily create 3D microstructures with various shapes depending on the shape of the microplates^12^. In this research, we produced a dodecahedron that consists of 12 microplates. The distance between each microplate was 5 μm, and the length of the microplate was 50 μm.

### Cell culture

NIH/3T3 cells (TKG299, RIKEN Cell Bank, Japan) and HepG2 cells (TKG0205, RIKEN Cell Bank, Japan) were cultured in High Glucose Dulbecco’s modified Eagle’s medium (HG-DMEM, Sigma-Aldrich, USA) with 10% fatal bovine serum (FBS, Sigma-Aldrich, USA) and 1% penicillin-streptomycin solution (P/S, Sigma-Aldrich, USA) in 60 mm dishes (Nunclon Delta Surface, Thermo Fisher, USA). Cells were cultured at 37 °C with 5% CO_2_.

Before culturing the cells, 20 µg/ml fibronectin (Funakoshi, Japan) was coated on the microplates to enhance cell adhesion (Supplementary Figure 2a). We prepared NIH/3T3 suspensions at different concentrations (2 × 10^5^ cells/ml, 3 × 10^5^ cells/ml, 4 × 10^5^ cells/ml and 5 × 10^5^ cells/ml) to determine *C*_N_ required to form 3D microstructures. NIH/3T3 cell suspension (1 mL) was seeded on the glass substrate with the microplates in a 40 mm non-adherent culture dish. We decided the culturing time of the cells was limited in 24 hours to speed up of a whole process of forming 3D microstructures. Non-adherent cells were washed and removed by changing medium after 4 h of cultivation. HepG2 cell suspension (1 mL) with *C*_H_ of 1 × 10^5^ cells/ml, 5 × 10^5^ cells/ml or 2.5 × 10^6^ cells/ml was then seeded on NIH/3T3 cells after 24 h (Supplementary Figure 2b). Non-adhered HepG2 cells were also washed and removed after 4 h of cultivation (Supplementary Figure 2c).

### Detachment and folding of the NIH/3T3-HepG2 cells

After 4 h cultivation of HepG2 cells, alginate lyase (Sigma-Aldrich, USA) at 40 μg/ml concentration was used for degrading the sacrificial layer of the alginate under the microplates, which triggered the self-folding process (Supplementary Figure 2d,e).

### Observation of cell morphology

To observe the co-cultured cells inside of the 3D microstructures, NIH/3T3 and HepG2 cells were stained with CellTracke Green CMFDA Dye and CellTracke Red CMTPX Dye (Invitrogen, USA), respectively, before seeding on the microplates. After formation of the 3D microstructures, the cells were observed by a fluorescence microscope (DP 72, Olympus, Japan) and a confocal microscope (Digital Eclipse C1, Nikon, Japan).

We used the LIVE/DEAD Viability/Cytotoxicity fluorescent imaging kit (Invitrogen, USA) to observe the viability of co-culture cells inside of the 3D microstructures from 1 to 6 days after formation of the microstructures. Living and dead cells were distinguished by green and red colours, respectively. The cells were dyed just before image acquisition by the confocal microscope.

### Measurement of albumin secretion

We evaluated the function of HepG2 cells by measuring the levels of secreted albumin in the folding group and the unfolding group. Two dishes of each group were prepared for each time point and the experiment was performed three times. The medium was changed every 24 h. From the 1^st^ to the 11^th^ day, the medium was collected, and albumin levels were measured using the albumin ELISA assay kits (EA3201-1, ASSAYPRO). The cell numbers of each dish were also counted and the amount of albumin per 10^4^ cells in each dish was determined.

## Electronic supplementary material

Supplementary Information

Supplementary movie 1

Supplementary movie 2
