# Supplementary material for: Origami-based self-folding of co-cultured NIH/3T3 and HepG2 cells into 3D microstructures
Source: Sci Rep. 2018 Mar 14;8:4556. doi: 10.1038/s41598-018-22598-x (PMC5852161; doi:10.1038/s41598-018-22598-x)
Supplement: Supplementary file 1 — Supplementary Information [file 41598_2018_22598_MOESM1_ESM.docx]

**Origami-based self-folding**

**of co-cultured NIH/3T3 and HepG2 cells**

**into 3D microstructures**

Qian He^1^, Takaharu Okajima^2^, Hiroaki Onoe^3^, Agus Subagyo^4^,

Kazuhisa Sueoka^2^ and Kaori Kuribayashi-Shigetomi^1^*

^1^ Institute for the Advancement of Higher Education, Hokkaido University, Sapporo, Japan

^2^ Graduate School of Information Science and Technology, Hokkaido University, Sapporo, Japan

^3^ Department of Mechanical Engineering, Faculty of Science and Technology, Keio University, Tokyo, Japan

^4^ Creative Research Institution Sousei, Hokkaido University, Sapporo, Japan

* Corresponding author: kaorik@ist.hokudai.ac.jp


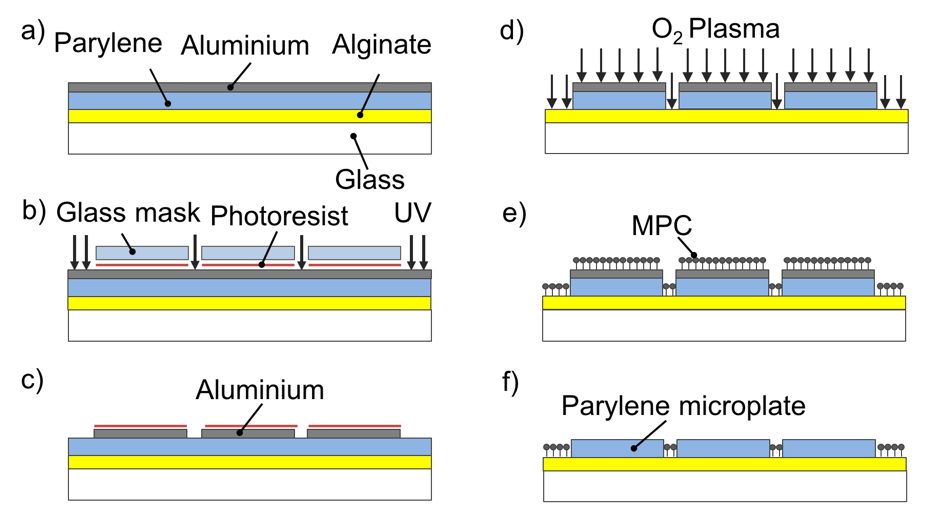


**Supplementary Figure 1.** The process of producing the microplates with the alginate layer. a) The alginate was coated on the glass substrate followed by parylene and aluminium deposition. b-c) After photoresist coating, the shape of the microplates can be patterned by UV exposure and aluminium etching. d) After the exposed parylene was removed by O_2_ plasma, the shape of the microplates was revealed. e) MPC polymer was coated on the entire surface of the glass substrate. f) Finally, the aluminium with the MPC polymer on the microplates was removed to reveal the final parylene microplate.


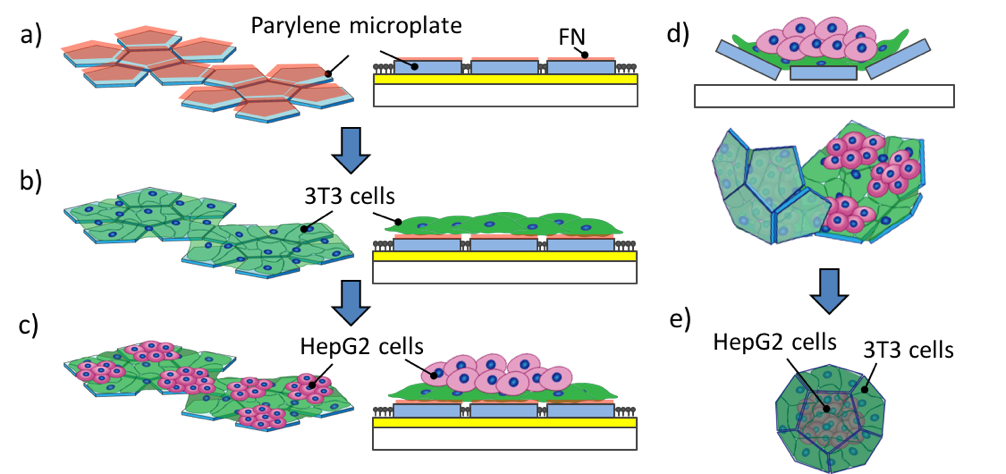


**Supplementary Figure 2**. Co-culture in folding and unfolding groups. a) Pentagon-shaped microplates were produced by the microfabrication technique. b-c) NIH/3T3 cells were seeded and cultured on the microplates for 24 h followed by HepG2 seeding. d-e) Alginate lyase removes the superficial layer, allowing the co-culture cells on the microplates to form a 3D microstructure.

**O****ptimization of initial cell seeding concentration of HepG2 cells**

We performed experiments to detect the secreted albumin from both folding and unfolding groups, using 1×10^5^ cells/ml, 5×10^5^ cells/ml and 2.5×10^6^ cells/ml as *C*_H_. The HepG2 cells could not be fully wrapped inside the 3D microstructures when using 2.5×10^6^ cells/ml; therefore, *C*_H_ should be less than 2.5×10^6^ cells/ml.


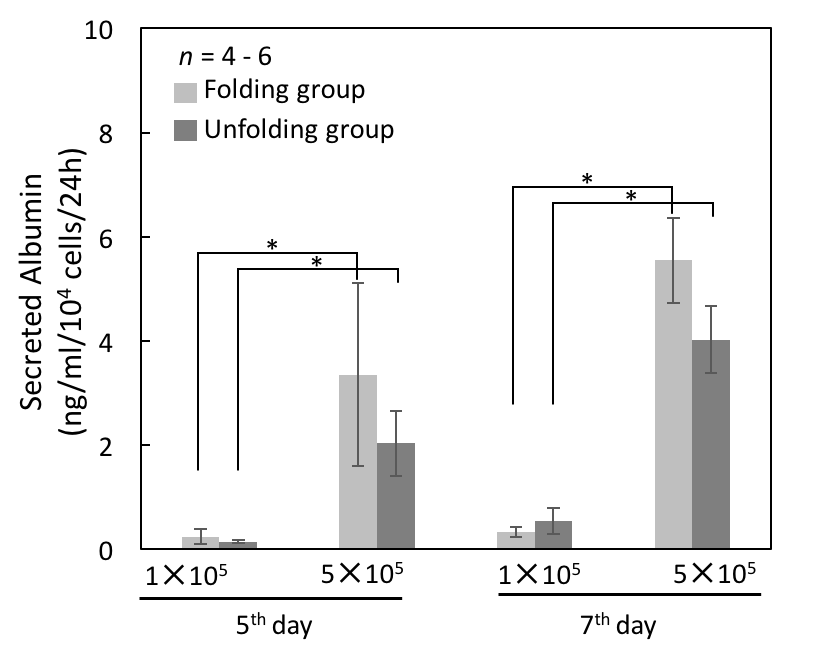


**Supplementary Figure 3**. Secreted albumin from co-culture cells with different *C*_H_. The secreted albumin in both groups was much lower when using 1×10^5^ cells/ml than when using 5×10^5^ cells/ml as *C*_H_, and the significant difference in folding and unfolding groups was found on both the 5^th^ and 7^th^ days.
